# Digital cognitive behavioral therapy versus Education for chronic pain in adults with sickle cell disease: 12-month randomized trial results

**DOI:** 10.1093/abm/kaag030

**Published:** 2026-07-21

**Authors:** Olubusola B Oluwole, Yi-Fan Chen, Christina M Lalama, Julia A O’Brien, Laura M De Castro, Charles Patrick Carroll, Darla Liles, Nirmish Shah, Santosh L Saraf, Victor R Gordeuk, Leshana Saint Jean, Michael DeBaun, Robert M Cronin, Tonya M Palermo, Sherif M Badawy, Megan E Hamm, Jennifer N Stinson, Chitra Lalloo, Sophie M Lanzkron, Cassandra Trimnell, Lakiea Bailey, Raymona H Lawrence, Kaleab Z Abebe, Charles R Jonassaint

**Affiliations:** Department of Medicine, University of Pittsburgh, Pittsburgh, PA 15213, United States; Hemophilia Center of Western PA, Pittsburgh, PA 15213, United States; Department of Medicine, University of Pittsburgh, Pittsburgh, PA 15213, United States; Center for Biostatistics and Qualitative Methodology, University of Pittsburgh, Pittsburgh, PA 15213, United States; Department of Medicine, University of Pittsburgh, Pittsburgh, PA 15213, United States; Center for Biostatistics and Qualitative Methodology, University of Pittsburgh, Pittsburgh, PA 15213, United States; Department of Acute and Tertiary Care, School of Nursing, University of Pittsburgh, Pittsburgh, PA 15261, United States; Department of Medicine, University of Pittsburgh, Pittsburgh, PA 15213, United States; Department of Psychiatry and Behavioral Sciences, Johns Hopkins University School of Medicine, Baltimore, MD 21287, United States; Division of Hematology-Oncology, East Carolina University, Brody School of Medicine, Greenville, NC, United States; Department of Medicine, Duke University, Durham, NC 27710, United States; Department of Medicine, Division of Hematology/Oncology, University of Illinois Chicago, Chicago, IL 60612, United States; Department of Medicine, Division of Hematology/Oncology, University of Illinois Chicago, Chicago, IL 60612, United States; Department of Pediatrics, School of Medicine, Vanderbilt University, Nashville, 37232, United States; Department of Pediatrics, School of Medicine, Vanderbilt University, Nashville, 37232, United States; Department of Medicine, The Ohio State University, Columbus, OH 43210, United States; Department of Anesthesiology & Pain Medicine, University of Washington & Seattle Children’s Research Institute, Seattle, WA 98105, United States; Department of Pediatrics, Northwestern University Feinberg School of Medicine, Chicago, IL 60611, United States; Division of Hematology, Oncology and Stem Cell Transplantation, Ann & Robert H. Lurie Children’s Hospital of Chicago, Chicago, IL 60611, United States; Department of Medicine, University of Pittsburgh, Pittsburgh, PA 15213, United States; Center for Biostatistics and Qualitative Methodology, University of Pittsburgh, Pittsburgh, PA 15213, United States; Lawrence Bloomberg Faculty of Nursing, University of Toronto, Toronto, ON M5T 1P8, Canada; Child Health Evaluative Sciences, Research Institute, The Hospital for Sick Children, Toronto, M5G 0A4, Canada; Institute of Health Policy, Management and Evaluation, University of Toronto, Toronto, M5T 3M6, Canada; Child Health Evaluative Sciences, Research Institute, The Hospital for Sick Children, Toronto, M5G 0A4, Canada; Institute of Health Policy, Management and Evaluation, University of Toronto, Toronto, M5T 3M6, Canada; Department of Medicine, Sidney Kimmel College of Medicine, Thomas Jefferson University, Philadelphia, PA 19107, United States; Sickle Cell 101, San Jose, CA 95110, United States; Sickle Cell Community Consortium, Cumming, GA 30040, United States; Foundation for Sickle Cell Disease Research, Hollywood, FL 33023, United States; Department of Medicine, University of Pittsburgh, Pittsburgh, PA 15213, United States; Center for Biostatistics and Qualitative Methodology, University of Pittsburgh, Pittsburgh, PA 15213, United States; Department of Medicine, University of Pittsburgh, Pittsburgh, PA 15213, United States; Department of Medicine, Emory University, Atlanta, GA 30322, United States

**Keywords:** sickle cell disease, pain, depression, eHealth, CBT, cognitive behavioral therapy

## Abstract

**Background:**

Chronic pain affects up to 40% of adults with sickle cell disease (SCD), yet treatment options remain limited. While cognitive behavioral therapy (CBT) is effective in other chronic pain conditions, it is underutilized for SCD pain, and its effectiveness remains unclear.

**Purpose:**

The Cognitive Behavioral Therapy and Real-time Pain Management Intervention for Sickle Cell via Mobile Applications (CaRISMA) compared digital CBT to Education for chronic SCD pain in adults. At 6 months, the primary outcome (pain interference) showed no between-group difference but both groups improved (CBT: −2.13; Education: −2.66). This report presents 12-month outcomes.

**Methods:**

A total of 359 participants with SCD chronic pain were randomized to 12 weeks of digital CBT (*n* = 181) or Education (*n* = 178), both with weekly health coach support.

**Results:**

At 12 months, 56.5% (*n* = 203) completed follow-up. No between-group differences in pain interference were observed [0.43, 95% CI, −1.61 to 2.48; *P* = .68]; however, both groups sustained improvement from baseline (CBT: −1.50; Education: −1.93). Cognitive behavioral therapy participants reported greater improvement in emotional impact [2.00, 95% CI, 0.12-3.88; *P* = .04], while pain intensity, depression, anxiety, and opioid misuse did not differ between groups. Higher health coach engagement was associated with reduced pain interference [−0.073 per 10% increase in engagement; *P* < .01].

**Conclusions:**

Although no between-group differences were found, both groups showed sustained improvement in pain interference at 12 months. Personalized support may have contributed to these improvements, highlighting the value of human support within digital interventions. Further research is needed to clarify the relative contributions of digital CBT vs human support.

**Study registration:**

This trial was registered on *ClinicalTrials.gov* (Identifier: NCT04419168. Registered 06/05/2020).

**Analytic plan registration:**

The trial protocol and analytic plan were pre-registered and are publicly available (https://www.researchprotocols.org/2021/5/e29014).

## Introduction

Sickle cell disease (SCD) disproportionately affects people of African descent and those from lower income backgrounds, yet these populations remain underrepresented in clinical research and trials^1^ particularly in studies addressing chronic pain,^2^^,^^3^ contributing to persistent gaps in access to evidence-based care.^4^ Pain in SCD is complex and can present as acute vaso-occlusive pain episodes (VOEs) or chronic pain. Chronic pain in SCD is increasingly recognized as a distinct clinical entity, separate from VOEs^5^ and is reported in up to 40% of adolescents and adults with SCD, commonly defined as persistent pain on most days for at least 6 months.^5^ Chronic pain in SCD is likely multifactorial and associated with central sensitization, psychological components (eg, depression and anxiety), sleep disturbance, increased healthcare utilization and reduced quality of life (QoL).^5^^,^^6^ While acute VOEs can often be managed with immediate analgesia (eg, high-dose opioids) and hydration, chronic pain is more challenging to treat.^7^^,^^8^ Long-term opioids carry unwanted side effects and additional psychological stressors for patients, who often face stigma.^6^ Psychological factors, including depression and anxiety, play a significant role in both acute and chronic pain.^9–12^ Although pharmacologic therapy (non-opioid and opioid analgesia) remains central to acute pain management, recent guidelines increasingly emphasize a multimodal, biopsychosocial approach to chronic pain that integrates non-pharmacologic strategies (eg, behavioral and other supportive interventions) alongside medications.^5^

There are significant racial and socioeconomic disparities in the management of chronic pain.^13–15^ Individuals with SCD face well-documented disparities in pain assessment and treatment, including delayed analgesia, undertreatment of pain, and stigmatization within healthcare settings.^4^^,^^6^^,^^16^^,^^17^ These disparities extend to access to non-pharmacologic pain interventions, which are less frequently offered or studied in this population despite evidence of benefit in other chronic pain conditions. Digital cognitive behavioral therapy (CBT) is becoming more widespread and can potentially improve access for all groups;^18^^,^^19^ however, limited trial data exist in underserved populations to confirm its effectiveness and identify optimal implementation strategies in these groups. Digital CBT, delivered via computer or mobile device, can be as effective as face-to-face therapy and, when combined with decentralized personal support, offers a scalable alternative to traditional therapy.^20^^,^^21^ While evidence from other chronic pain populations is promising, CBT implementation for adults with SCD has been limited.^22^^,^^23^ Recently, the first adequately powered trial of CBT in SCD^24^ demonstrated that digital CBT improved pain outcomes in adolescents at 6 months compared to an education control. The Cognitive Behavioral Therapy and Real-time Pain Management Intervention for Sickle Cell via Mobile Applications (CaRISMA) trial then extended these findings to adults by comparing tailored digital CBT to a digital pain and SCD education program, both supported by health coaches.^25^ While the 6-month results showed no between-group differences in pain interference, both arms demonstrated improvement in pain interference.^26^ This may be due to health coach support on both arms. However, more data on the durability of digital interventions with this population is needed to understand the overall clinical implications of recent findings and to guide future behavioral trials with underserved populations. Prior studies of both in-person and digital CBT across chronic pain, depression, and anxiety demonstrate that benefits in pain, functional, and emotional outcomes can persist beyond 6 months;^20^^,^^27–31^ however, long-term outcomes have not been evaluated in adults with SCD. Longer follow-up is particularly important when comparing 2 active interventions, as early improvements may reflect shared attention or support effects rather than sustained differences in skill use. Meta-analyses indicate that CBT effects may attenuate over time when compared to active controls,^32^ and some trials suggest that CBT-related improvements in psychological outcomes may be more durable than education alone.^15^^,^^33^ Because both CaRISMA arms included active digital content and health coach support, the lack of between-group differences at 6 months did not exclude the possibility that differences could emerge or change over time. Given the clinical importance of durability in chronic SCD pain and the absence of long-term data in this population, we examined CaRISMA outcomes at 12 months.

## Methods

### Study design

The Cognitive Behavioral Therapy and Real-time Pain Management Intervention for Sickle Cell via Mobile Applications was a multi-site, randomized, pragmatic, comparative effectiveness trial conducted at 7 comprehensive sickle cell centers. Additional recruitment was also done through 3 community-based organizations (CBOs) and their partners, where patients were recruited via the web, social media, and in-person at community events and meetings. Eligible patients were identified using screening tools, best practice alerts, and study/CBO websites. Patients were recruited from August 19, 2020 to December 04, 2022, during the COVID pandemic. Enrolled participants were randomized 1:1 to receive either (1) digital CBT intervention tailored for adults with SCD (digital CBT arm) or (2) SCD and pain education on their mobile phone (Education arm) using permuted block allocation stratified by site. The digital CBT intervention focused on teaching behavioral coping skills through a “seeing and doing” approach, while the Education arm aimed to improve self-management through a “learning and knowing more about SCD and pain” approach. Both arms had access to a health coach who provided personalized support and motivation to engage with the interventions. During the study period, healthcare utilization was abstracted from the electronic medical records to measure emergency department (ED) visits, hospitalizations, and opioid prescriptions for participants in the CBT and Education groups.

The study was approved by the Institutional Review Board and all participants provided written informed consent. Detailed study description and methods are published in the trial protocol.^25^ The trial is registered on ClinicalTrials.gov (NCT04419168).

### Participants

Inclusion criteria for the CaRISMA trial were individuals with SCD who (1) were able to speak/understand English, (2) were ≥ 18 years old, (3) reported chronic pain, defined as pain at least 4 days a week over the past 3 months or longer, and/or being prescribed long-acting or daily opioid medication for pain, and (4) already owned or were willing to be provided a smartphone. For participants enrolled virtually, self-reported SCD was accepted at the time of enrollment, with medical documentation of SCD status obtained subsequently. Participants with cognitive dysfunction or low literacy, identified by failing a 6-item consent comprehension assessment or individuals who were not willing to participate in an intervention arm of the study were excluded.

### Interventions

The study interventions were delivered by a scripted chatbot, which was accessed through the Facebook Messenger app. Health coaches communicated with participants by text messages or phone calls. The chatbot was developed collaboratively with the majority of content being developed using human-centered design. We conducted group ideation sessions with patients, family members, providers, and researchers to identify critical topics, relevant narratives, and applicable skills. The study team then drafted scripts based on these findings, which stakeholders reviewed in subsequent sessions to ensure language resonated with the target population. Video actors, all adults with SCD, further adapted scripts to reflect their authentic voices. A CBT-trained psychologist participated in all sessions to ensure fidelity to evidence-based principles.

The resulting chatbot offered tailored responses, educational materials, and motivational content in various formats including videos, GIFs, and images, all adapted to participants’ inputs.

#### Digital CBT

The digital CBT arm targeted multiple core cognitive and behavioral pain coping skills, including cognitive restructuring, behavioral activation, relaxation strategies, goal setting, and problem-solving. It emphasized skills acquisition through practice, homework assignments, challenges, and check-ins with a health coach. Participants also had access to a study-associated Facebook page for peer support.

#### Education

The digital Education arm focused on pain and SCD education, teaching users about chronic pain, the physiology and neuroscience underpinning SCD pain, healthy lifestyle tips, and facts about SCD including genetic inheritance. It emphasized knowledge acquisition through brief quizzes and discussion with the health coach and their social network.

#### Health coach

Human support is not universally required for digital interventions to be effective; however, evidence suggests that the presence and intensity of support may meaningfully influence engagement, adherence, and clinical outcomes depending on the population, condition, and intervention design.^34^ In this trial, health coaches provided emotional support through reflective listening and informational guidance through weekly sessions with participants, conducted by phone or text message when calls were not feasible or preferred. While health coaches did not deliver CBT or pain education directly, they encouraged participants to engage with the chatbot content.

Health coaches were volunteers from the SCD community (patients, advocates, and family members) with college-level education or higher. Coaches completed 8 hours of training and attended weekly supervision with a Masters-level psychologist or behavioral specialist and the principal investigator to review interactions, solve problems, and maintain coaching quality. Text communications between coaches and participants were monitored to ensure protocol fidelity.

### Outcomes and measures

Most of the outcome measures were collected at baseline, and at 3-, 6-, and 12-month follow-ups unless otherwise specified.

#### Pain interference

This was measured by the 8-item Patient-Reported Outcomes Measurement Information System Pain Interference (PROMIS-8a)^35^ which measures pain impact related to social, cognitive, emotional, physical, and recreational activities. Participants respond using 5-point Likert scales with higher scores indicating greater pain interference. Raw total scores (ranging from 8 to 40) are transformed into T-scores with mean = 50, SD = 10.

#### Pain intensity

Participants were asked to record daily pain scores (on a 0-10 scale), opioid usage, and mood using a mobile web app. They received daily reminders via text for the 2 weeks after each follow-up time point. Mean pain intensity during each 2-week assessment period was used for analyses.

#### Depression

Depression was assessed using a stepped screening approach designed to reduce participant burden and ensure appropriate management of suicide risk in this remote trial. All participants first completed the Patient Health Questionnaire-2 (PHQ-2),^36^ which assesses depressed mood and anhedonia, the cardinal symptoms required for a depressive disorder. Participants scoring 0 on the PHQ-2 were classified as having no significant depressive symptoms and did not complete additional items. Those scoring >0 completed full PHQ-8 (community/virtual participants) or PHQ-9 (clinical site participants). Both measures used a 0-3 scale per item, with higher scores indicating greater depression severity: 5 (mild), 10 (moderate), 15 (moderately severe), and 20 (severe).^37^ For consistency across the sample, all analyses used PHQ-8 total scores (range 0-24)

#### Anxiety

Similar to depression, a stepped screening approach was used for anxiety. Symptoms were assessed using the Generalized Anxiety Disorder scale (GAD-2 and GAD-7).^38^ The full GAD-7 items were administered to participants scoring >0 on GAD-2. Scores range from 0 to 21, with higher scores indicating greater severity: 5 (mild), 10 (moderate), and 15 (severe).

#### Opioid misuse

The Current Opioid Misuse Measure (COMM-9),^39^ a 9-item self-assessment tool using a 5-point Likert scale, evaluated opioid-related behaviors over the previous 30 days. Higher scores indicate greater risk of misuse. Opioid misuse data were collected at baseline and 12 months.

#### Quality of life

The Adult Sickle Cell Quality of Life Measurement Information System (ASCQ-Me) assessed social functioning and emotional distress domains, with higher scores indicating worse SCD-related QoL.^40^

### Statistical analysis

While the study was powered for the primary outcome at 6 months, 12-month analyses were prespecified as secondary and exploratory.^26^ Descriptive statistics, including means and SDs for continuous variables and sample proportions for categorical variables, were calculated for all variables. Intention-to-treat analyses were conducted using linear mixed models with time, study arm, their interaction, study site, and baseline depression level as fixed effects, and subject-level random effects. Contrasts assessed the impact of the interventions on 12-month improvements. For the primary outcome, determinants of missing data were evaluated by comparing attrition rates between study arms and baseline characteristics of participants with and without the pain interference missing at 12 months. A joint model as a sensitivity analysis thus assessed the robustness of the intervention effect assuming non-ignorable missing. This joint model comprised the previously described linear mixed model for the primary outcome and a logistic mixed model, which adjusted for significant factors found in the missing mechanism, for any dropout or missing with a shared random intercept.

Exploratory analyses investigated the impact of intervention engagement on pain interference by restricting to participants who started at least 1 lesson and conducting “intensity-adjusted” analyses based on the proportion of completed chatbot sessions and health coach interactions.

## Results

**Figure 1:**
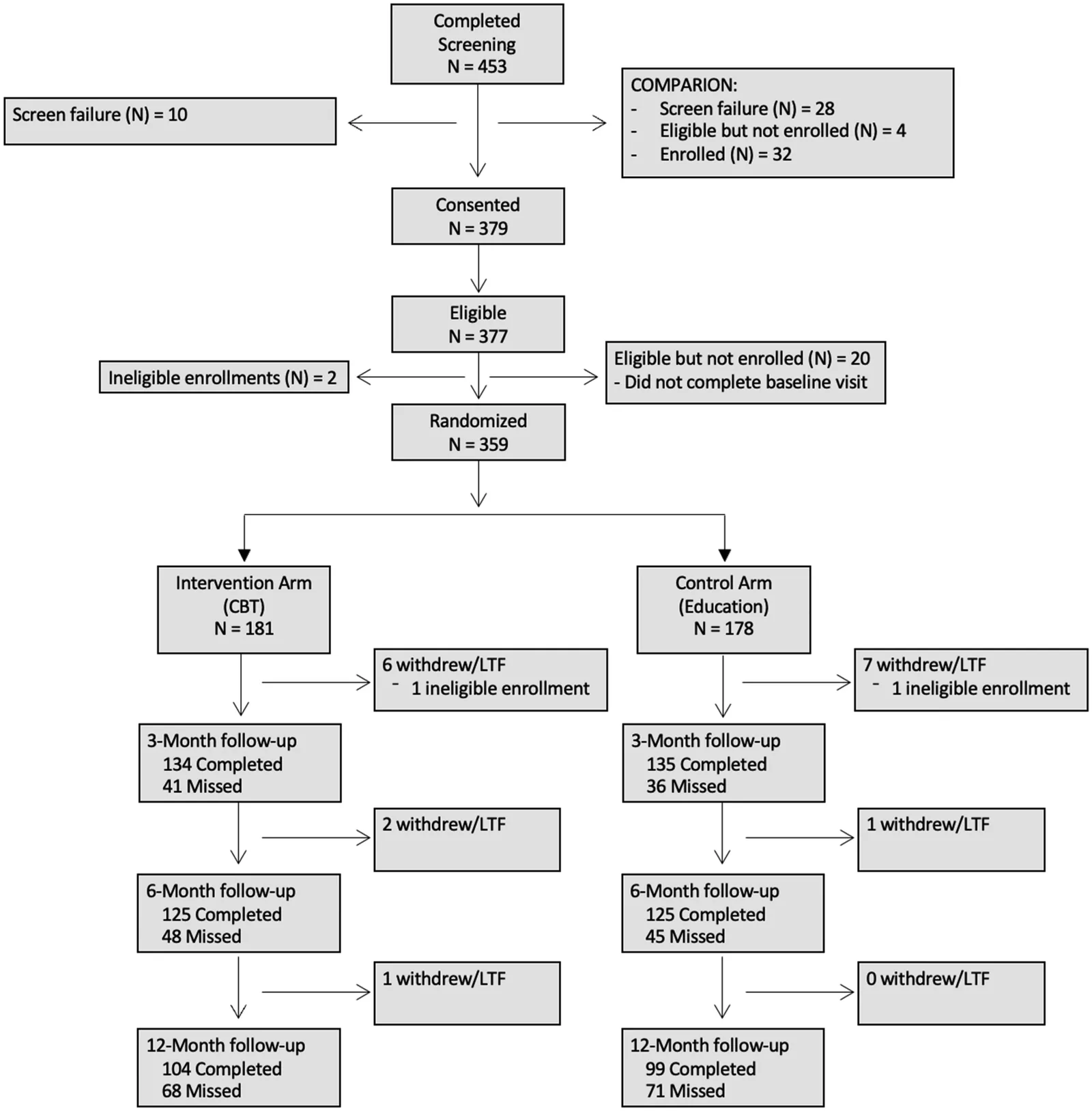
Participant flow through the study (CONSORT diagram).

Figure 1 shows the CONSORT diagram of participants through different stages of the study. A total of 453 individuals completed screening, 359 (79.2%) were randomized to receive either digital CBT (*n* = 181) or Education (*n* = 178). A total of 125 participants in each group completed the 6-month visit. By 12 months, 104 (57.4%) participants had completed follow-up in the digital CBT group and 99 (55.6%) in the Education group. Withdrawal/lost-to-follow-up rates were similar between both groups (CBT: *n* = 77, 42.5%; Education: *n* = 79, 44.4%, *P* = .73).

### Patient characteristics

Demographics were similar across arms, Table 1. Mean age was 36.4 years (SD = 9.9) for CBT (*n* = 181) and 36.3 years (SD = 11.1) for Education (*n* = 178). Women represented 61.3% (*n* = 111) of the CBT group and 71.9% (*n* = 128) of Education, with most participants identifying as Black/African American (CBT: 93.4%, *n* = 169; Education: 91.6%, *n* = 163). Of the 359 participants 265 (73.8%) were enrolled in clinic, with 94 (26.2%) enrolled virtually. Educational attainment was comparable, with roughly 75% in both groups having at least some college. About 45% of participants (CBT: 45.9%, *n* = 83; Education: 45.5%, *n* = 81) were on disability, while 37% (CBT: 37%, *n* = 67; Education: 36.5%, *n* = 65) were employed. Nearly all participants (96.4%, *n* = 346) had confirmed SCD diagnosis.

**Table 1:** Participant demographics by randomization arm.

Characteristic	CBT (*N* = 181)	Education (*N* = 178)	Total (*N* = 359)
**Age at baseline, mean (SD)**	36.4 (9.9)	36.3 (11.1)	36.3 (10.5)
**Gender, F (*n*, %)**	111 (61.3%)	128 (71.9%)	239 (66.6%)
***Enrolled via:***			
** Clinic**	133 (73.5%)	132 (74.2%)	265 (73.8%)
** Virtual**	48 (26.5%)	46 (25.8%)	94 (26.2%)
**Confirmed SCD diagnosis**	176 (97.2%)	170 (95.5%)	346 (96.4%)
**Race (Black/African American)**	169 (93.4%)	163 (91.6%)	332 (92.5%)
***Education attainment***			
** Some high school**	12 (6.6%)	10 (5.6%)	22 (6.1%)
** High school or equivalent**	33 (18.2%)	36 (20.2%)	69 (19.2%)
** Some college**	83 (45.9%)	77 (43.3%)	160 (44.6%)
** Completed college**	31 (17.1%)	30 (16.9%)	61 (17.0%)
** Graduate**	22 (12.2%)	25 (14.0%)	47 (13.1%)
***Employment status***			
** Employed**	67 (37.0%)	65 (36.5%)	132 (36.8%)
** Not employed**	31 (17.1%)	32 (18.0%)	63 (17.5%)
** On disability**	83 (45.9%)	81 (45.5%)	164 (45.7%)

### Patient characteristics of those who completed pain interference questionnaires at 12 months

We next examined the baseline characteristics associated with 12-month follow-up completion. Women were significantly more likely to complete the 12-month pain interference assessment (77.2% vs. 52.9% in those who did not complete, *P* < .01, Table 2). Completers were slightly older (mean age 37.1 vs 35.4 years, *P* = .03). Employment status was different between completers and non-completers (*P* < .01) with completers less likely to be employed (30.2% vs 45.2%) and more likely to be on disability (56.4% vs 31.8%). Completers also had lower baseline anxiety scores on the GAD-7 (mean 8.0 vs 9.0, *P* = .04) and higher diary completion rates during the baseline assessment period (10.0 vs 7.0 entries, *P* < .01). There were no significant differences in baseline pain interference, pain intensity, depression scores, and QoL between completers and non-completers.

**Table 2:** Participant demographics by completion status of 12-month pain interference data.

	Non-completers (*n* = 157)	Completers (*n* = 202)	*P*-value^a^
***Randomized arm***			.83
** CBT**	78 (49.7%)	103 (51.0%)	
** Education**	79 (50.3%)	99 (49.0%)	
***Enrolled via:***			1.00
** Clinic**	116 (73.9%)	149 (73.8%)	
** Virtual**	41 (26.1%)	53 (26.2%)	
**Age at baseline, mean (SD)**	35.4 (11.1)	37.1 (10.0)	.03
**Sex**			<.001
** Male**	74 (47.1%)	44 (21.8%)	
** Female**	83 (52.9%)	156 (77.2%)	
***Educational attainment***			.34
** Some high school**	12 (7.6%)	10 (5.0%)	
** High school or equivalent**	36 (22.9%)	33 (16.3%)	
** Some college**	63 (40.1%)	97 (48.0%)	
** Completed college**	27 (17.2%)	34 (16.8%)	
** Graduate**	19 (12.1%)	28 (13.9%)	
***Employment status***			<.001
** Employed**	71 (45.2%)	61 (30.2%)	
** Not employed**	36 (22.9%)	27 (13.4%)	
** On disability**	50 (31.8%)	114 (56.4%)	
**Pain interference, mean (SD)**	61.9 (7.1)	63.1 (7.1)	.11
**Number of pain diary entries, mean (SD)** ^b^	7.0 (4)	10.0 (4)	<.001
**Pain intensity, mean (SD)** ^c^	4.2 (2.7)	4.5 (2.4)	.41
**Depression (PHQ score), mean (SD)** ^d^	10.2 (4.8)	10.0 (5.1)	.64
**Anxiety (GAD-7 score), mean (SD)**	9.0 (4)	8.0 (5)	.042
**Quality of life: social functioning, mean (SD)**	47.5 (7.7)	47.2 (8.0)	.72
**Quality of life: emotional impact, mean (SD)**	47.9 (9.0)	48.1 (8.7)	.78

a *T*-test or Wilcoxon test, as appropriate, for continuous variables and Fisher’s exact test for categorical variables.

b Pain diary entries during the 2-week reminder period.

c Pain Intensity average during the 2-week reminder period.

d For all participants with baseline PHQ-2 > 0, all depression severity analyses were conducted using PHQ-8 scoring for comparability across participants.

### Primary between-group analyses

Primary between-group analyses (Table 3) showed no statistically significant difference in pain interference at 12 months, the primary outcome, between the CBT and Education groups [mean difference = 0.43, 95% CI, −1.61 to 2.48; *P* = .68] and a similar result was observed in the sensitivity analysis assuming non-ignorable missing (data not shown). Similarly, there were no between-group differences in secondary outcomes including pain intensity [0.09, 95% CI, −0.46 to 0.63; *P* = .75], depression (PHQ-8: −1.23, 95% CI, −2.62 to 0.16; *P* = .08), anxiety (GAD-7: −0.24, 95% CI, −1.69 to 1.21; *P* = .75), opioid misuse (COMM-9: −0.34, 95% CI, −1.51 to 0.82; *P* = .56), and QoL-social functioning (ASCQ-Me: 0.71, 95% CI, −1.30 to 2.71; *P* = .49). However, a significant between-group difference was observed in emotional QoL, with greater improvement in the CBT group [2.00, 95% CI, 0.12-3.88; *P* = .04; Cohen’s *d* = 0.22)].

**Table 3:** Between-group changes of primary and secondary outcomes at 12-months.

Outcome	CBT mean (SD)^a^	Education mean (SD)^a^	Between-group difference mean change (95% CI)^b^	*P*-value^b^	Cohen’s *d* (95% CI)
**Pain interference (PROMIS-8a)**	61.0 (8.4)	61.0 (7.9)	0.43 (−1.61, 2.48)	.68	0.04 (−0.16, 0.25)
**Pain intensity (0-10)**	4.1 (2.7)	4.4 (2.8)	0.09 (−0.46, 0.63)	.75	0.03 (−0.18, 0.25)
**Depression (PHQ-8)** ^c^	9.8 (4.5)	9.0 (4.7)	−1.23 (−2.62, 0.16)	.08	−0.20 (−0.43, 0.03)
**Anxiety (GAD-7)** ^d^	8.0 (4.3)	7.8 (4.7)	−0.24 (−1.69, 1.21)	.75	−0.04 (−0.29, 0.21)
**Opioid misuse (COMM-9)**	7.5 (5.0)	6.9 (5.0)	−0.34 (−1.51, 0.82)	.56	−0.06 (−0.27, 0.15)
**Social functioning (ASCQ-Me)**	49.4 (8.8)	48.8 (7.6)	0.71 (−1.30, 2.71)	.49	0.07 (−0.13, 0.28)
**Emotional impact (ASCQ-Me)**	50.0 (8.0)	50.3 (7.7)	2.00 (0.12, 3.88)	.04	0.22 (0.01, 0.43)

a Unadjusted raw means and SDs.

b From linear mixed models with time, study arm, their interaction, study site, and baseline depression level as fixed effects, and subject-level random effects.

c For all participants with baseline PHQ-2 > 0, all depression severity analyses were conducted using PHQ-8 scoring for comparability across participants.

d GAD-7 for all participants with baseline GAD-2 > 0.

### Exploratory within-group analyses

Although not a pre-specified outcome, we also examined within-group changes (Table 4). Both groups demonstrated within-group improvement in pain interference scores [CBT: −1.50, 95% CI, −2.94 to −0.07; Education: −1.93, 95% CI, −3.39 to −0.47]. For 0-10 daily pain intensity, there were no significant within-group differences. Depression scores also improved in both groups: [CBT group: −1.45, 95% CI, −2.45 to −0.44; Education group: −0.22, 95% CI, −1.18 to 0.75], as did opioid misuse scores [CBT: −1.72 (−2.54, −0.91); Education: −1.38 (−2.21, −0.55)]. Improvement in social functioning QoL, were also observed [CBT: 2.31, 95% CI, 0.91-3.72; Education: 1.61, 95% CI, 0.17-3.04].

**Table 4:** Within-group changes of primary and secondary outcomes at 12 months.

Assessment	Within-group difference (CBT) mean change (95% CI)^a^	Within-group difference (Education) mean change (95% CI)^a^
**Pain interference score**	−1.50 (−2.94, −0.07)	−1.93 (−3.39, −0.47)
**Pain intensity score**	0.06 (−0.31, 0.44)	−0.02 (−0.42, 0.37)
**Depression (PHQ-8)** ^b^	−1.45 (−2.45, −0.44)	−0.22 (−1.18, 0.75)
**Anxiety (GAD-7)** ^c^	−0.70 (−1.69, 0.29)	−0.46 (−1.52, 0.59)
**Opioid misuse (COMM-9)**	−1.72 (−2.54, −0.91)	−1.38 (−2.21, −0.55)
**Quality of life: social functioning**	2.31 (0.91, 3.72)	1.61 (0.17, 3.04)
**Quality of life: emotional impact**	3.03 (1.71, 4.34)	1.03 (−0.31, 2.37)

a From linear mixed models with time, study arm, their interaction, study site, and baseline depression level as fixed effects, and subject-level random effects.

b For all participants with baseline PHQ-2 > 0, all depression severity analyses were conducted using PHQ-8 scoring for comparability across participants.

c GAD-7 for all participants with baseline GAD-2 > 0.

### Impact of treatment engagement on pain interference outcomes at 12 months

The analysis of health coach engagement across groups demonstrated a significant association between increased completion of health coach sessions and reductions in pain interference at 12 months, as depicted Table 5. Specifically, for every 10% increase in completed sessions, there was a 0.73-point reduction in pain interference (95% CI, −1.12 to −0.34, *P* < .001).

**Table 5:** Twelve-month changes in pain interference relative to chatbot and health coach engagement in both arms.

Intervention type	% intervention completed					Estimate 95% CI^a^	*P*-value
	0%	25%	50%	75%	100%		
**Proportion chatbot lessons completed**	−1.19	−1.48	−1.78	−2.08	−2.38	−0.012 (−0.039, 0.015)	.39
**Proportion of HC sessions within 3 months of enrollment**	0.67	−1.14	−2.96	−4.77	−6.59	−0.073 (−0.112, −0.034)	<.01

a Estimated decline in pain interference at 12 months for each % increase in intervention completed from linear mixed models with time, engagement intervention, their interaction, study site, and baseline depression level as fixed effects, and subject-level random effects.

Abbreviation: HC = health coach. Values represent modeled change in pain interference T-score from baseline to 12 months at varying levels of intervention engagement. Negative values reflect improvement (ie, reduced pain interference).

Chatbot engagement and completion rates declined over time. Of all participants, 44/181 (24%) in the CBT arm and 41/178 (23%) in the Education arm never connected to the chatbot while an additional 35/181 (19%) and 16/178 (9%), respectively, connected but did not start any lessons. Among randomized participants, lesson completion declined quickly (Lesson 1 completed: 81/181 [45%] CBT vs 91/178 [51%] Education; Lesson 4: 36/181 [20%] CBT vs 57/178 [32%] Education) (data previously presented in our 6-month outcomes paper).^26^ The proportion of chatbot lessons completed was not significantly associated with 12‑month change in pain interference as 10% increase in completed section was associated with a decrease by 0.012 points ([95% CI, −0.039 to 0.015, *P* = .39], Table 5), although more attenuated than in the overall cohort.

## Discussion

In this 12-month follow-up of the CaRISMA study, there was no significant difference between groups in pain interference. Furthermore, there was no significant difference between groups in daily pain intensity, anxiety, depression, opioid misuse, and social functioning QoL. However, the CBT group showed greater improvement in emotional impact compared to the Education group, although the effect size was small (Cohen’s *d* = 0.22). Despite poor engagement with the digital component of the interventions, health coach support engagement was relatively high and was associated with greater reductions in pain interference. Lastly, as an exploratory outcome, both interventions showed sustained within-group improvements in pain interference over 12 months. Although the overall change was modest, the ability of digital behavioral interventions, when paired with personalized support, to yield long-term benefits without side effects, and at lower cost and greater scalability than face-to-face treatments, underscores their potential value in clinical settings.

To date, the CaRISMA trial represents one of the most comprehensive evaluations of digital CBT for a racial/ethnic minority population to date. With 359 participants followed for 12 months, this is the largest non-pharmacological trial in SCD and substantially expands previous research that was largely limited to pediatric populations or small, short-term studies in adults. To our knowledge, the only other adequately powered behavioral trial in SCD is Palermo et al.’s^24^ study examining digital CBT vs Education control in 137 adolescents ages 12-18 years. Both trials showed similar patterns in pain interference, with within-group improvements in the CBT arm but no significant between-group differences (*P* = .065).^26^ Palermo et al. demonstrated significant reductions in pain intensity and frequency among adolescents at 6-month follow-up (*d* = 0.50) whereas CaRISMA found no significant between-group differences in pain intensity among adults at 6^41^ and 12 months. This discrepancy may reflect higher baseline pain levels in the adult sample (∼4.2-4.5/10 vs ∼3.0/10 in adolescents),^24^^,^^26^ reflecting the progressive nature of SCD pain that often becomes more severe and chronic with age.

Additionally, both CaRISMA arms received health coach support during or soon after the COVID-19 pandemic, when social isolation was prevalent. This shared support during a period of heightened social isolation may have provided therapeutic benefit to both groups, making it difficult to isolate the specific effects of CBT and potentially reducing difference between groups. Further, there was an overrepresentation of women, who tend to report higher baseline pain^41^ and engage more with behavioral interventions,^42^^,^^43^ which may have influenced within-group improvements, though this effect is difficult to quantify.

Regardless of what drove these effects, both groups demonstrated consistent, durable improvements over 12 months. Participants began the study approximately 12-13 points above the general population mean on PROMIS Pain Interference (where 50 represents typical adult pain levels). The observed 2-point reduction represents roughly 15%-20% of the gap between baseline SCD pain levels and general population norms. Even this modest sustained improvement may have meaningful long-term effects on functioning, healthcare utilization, and QoL. The effect sizes observed likely represent the floor of what is possible; participants who completed all 12 health coach sessions demonstrated nearly a 7-point reduction. This suggests that with improved engagement and optimized delivery, behavioral interventions hold considerable promise. The next phase for this work should address how to effectively implement these interventions, overcome barriers to engagement, and reduce cost and burden for patients and providers.

### Psychological and emotional impact

Chronic pain often imposes significant psychological burden. We found a between-group difference in emotional impact suggesting that CBT may offer added benefits in addressing the emotional challenges associated with chronic pain beyond physical symptom management. Palermo et al.’s^24^ randomized controlled trial comparing CBT to education in children with SCD found no significant differences between the groups in depression and anxiety at 6 months. Similarly, we were not able to detect a group difference in depression and anxiety at 6 months,^26^ though there was a within-group reduction in depressive symptoms (PHQ-8) in both arms at 6 months which was sustained at 12 months. The CBT intervention was associated with a modest improvement in ASCQ-Me Emotional Impact scores over 12 months compared with the education group. This suggests that any CBT-related effects were limited to disease-related emotional coping rather than general psychiatric symptoms. The Emotional Impact scale captures emotional responses to living with SCD (eg, “I feel discouraged about my health”), whereas the PHQ and GAD assess broader psychiatric constructs. Given the small effect size, these findings should be interpreted with caution.

CaRISMA’s warrior stories and testimonials were designed to normalize and validate the sickle cell experience-content aligned with health-related QoL rather than targeted mental health treatment. Future interventions seeking to address comorbid depression and anxiety may need to incorporate more specific mental health components alongside disease management content, and future studies may benefit from examining the impact of peer-led behavioral interventions on health-related stigma.

### Health coach and intervention engagement

As previously presented,^26^ 79.7% of all participants completed at least one health coach session (CBT: 77%; Education: 82%). The mean number of sessions completed overall was 4.1 out of 12 (CBT: 4.1 (SD 2.8); Education 4.3 (SD 3.0). Most of these sessions were over the telephone with a mean duration of 27 and 26 minutes in the CBT and Education arm respectively. Although it is challenging to separate the effects of health coach vs CBT intervention, health coach engagement was significantly associated with reduced pain interference at 12 months in contrast to our results at 6 months. This delayed effect may reflect the cumulative nature of behavior change, where skills such as reframing and goal setting, introduced during the 12-week coaching period, require time and repeated practice to translate into sustained improvements. By providing emotional support through shared experiences and practical advice, health coaches provide an additional layer of engagement and support, likely enhancing overall outcomes.

Unfortunately, the study also identified a decline in engagement rates with each subsequent chatbot lesson, particularly in the CBT group. These findings are consistent with existing research on digital interventions, which often show reduced engagement as a common barrier to long-term efficacy.^24^^,^^44^ Strategies to boost engagement, such as personalized feedback or gamification, could potentially mitigate this issue. The relatively high proportion of participants in the CBT group who did not engage with the chatbot indicates a need for improved user experience and accessibility in digital interventions, particularly for populations with high rates of disability and chronic pain.

Overall, patient engagement in our study demonstrated both strengths and challenges compared to other decentralized digital trials that lack in-person contact. While automated reminders and a mobile pain-tracking platform facilitated participation, engagement fluctuated over time, particularly outside of structured reminder periods. Digital trials that incorporate real-time feedback, social support, or interactive components tend to report higher adherence rates, as seen in interventions utilizing chatbots or health coaching.^45^ For instance, a meta-analysis by Meyerowitz-Katz et al. reported an average attrition rate of 43% in app-based health interventions, highlighting significant dropout rates over time which was similar to the attrition rates in our study.^46^

### Strengths and limitations

This study has several strengths. First, the CaRISMA trial represents one of the largest, prospective studies examining the effects of digital CBT on pain management in adults with SCD, contributing valuable data to the field of non-pharmacological pain management. Second, the study involves a diverse group of participants recruited from both academic sickle cell centers as well as the community. Third, it involves a 12-month longitudinal follow up that allows for capturing of changes over time and obtaining data on the durability of intervention effects. Fourth, the study utilizes a scalable digital intervention that would be accessible to a diverse population and can be more easily integrated into routine care. Conversely, this study has limitations that should be considered when interpreting the results. Attrition rates, though similar between the CBT and Education groups and addressed in a sensitivity analysis, may have affected the overall outcomes. Engagement was lower than expected, particularly with the digital CBT intervention and chatbot lessons, potentially diluting the intervention’s effectiveness as participants may not have received the full benefit of the digital interventions. Participants were not trained on how to engage with the chatbot, as the intervention was designed to be self-guided; however, this lack of onboarding may have limited participants’ ability to effectively navigate and use the platform, contributing to low engagement and potentially attenuating the observed effects of the digital intervention. Lastly, because both study arms included health coach support, we were unable to isolate the independent effects of digital CBT content vs human support. The absence of an unsupported digital arm or a health-coach-only comparator limits causal inference regarding the specific contribution of CBT.

### Conclusion

The goal of this study was to examine long-term outcomes of the CaRISMA trial. Although digital CBT did not significantly outperform Education on most clinical outcomes, it was associated with greater improvement in emotional impact QoL among adults with SCD. The study also highlighted the critical role of health coach engagement in treatment outcomes, showing that active participation in health coach sessions was significantly associated with reductions in pain interference. Given that coaching was provided in both arms, these findings do not allow causal attribution to coaching vs digital content. Additionally, longer follow-up periods are needed to fully understand the long-term impact of CBT on pain management and psychological outcomes in SCD patients. Taken together, the results suggest that supported digital behavioral programs may yield durable, incremental benefits for chronic SCD pain, while highlighting the importance of study designs that can better determine the individual contributions of human support and digital intervention components and address barriers to long-term engagement.

## Data Availability

The data that support the findings of this study are available upon reasonable request from the corresponding author, in accordance with PCORI policies and IRB restrictions.

## Analytic code availability

Analytic code used for the statistical analyses is available upon request to the corresponding author.

## Materials availability

Intervention materials (ie, chatbot scripts, education modules,) are available upon request to the study team, subject to copyright and licensing restrictions.
